# The management of retrorectal tumors – a single-center analysis of 21 cases and overview of the literature

**DOI:** 10.1007/s00423-024-03471-0

**Published:** 2024-09-14

**Authors:** K. Fechner, B. Bittorf, M. Langheinrich, K. Weber, M. Brunner, R. Grützmann, K. E. Matzel

**Affiliations:** 1 Department of Surgery, Friedrich-Alexander-University Erlangen-Nuremberg (FAU), Krankenhausstraße 12, D-91054 Erlangen, Germany; 2 Department of General, Thoracic and Vascular Surgery, Greifswald University, Ferdinand-Sauerbruch-Straße, Greifswald, Germany; 3 Comprehensive Cancer Center Erlangen-EMN (CCC ER-EMN), Erlangen, Germany; 4 Comprehensive Cancer Center Alliance WERA (CCC WERA), Erlangen, Germany; 5 Bavarian Cancer Research Center (BZKF), Erlangen, Germany

**Keywords:** Retrorectal tumors, Tailgut cyst, Presacral mass, Treatment

## Abstract

**Aim:**

Retrorectal tumors are rare and heterogeneous. They are often asymptomatic or present with nonspecific symptoms, making management challenging. This study examines the diagnosis and treatment of retrorectal tumors.

**Methods:**

Between 2002 and 2022, 21 patients with retrorectal tumors were treated in our department. We analyzed patient characteristics, diagnosis and treatment modalities retrospectively. Additionally, a literature review (2002–2023, “retrorectal tumors” and “presacral tumors”, 20 or more cases included) was performed.

**Results:**

Of the 21 patients (median age 54 years, 62% female), 17 patients (81%) suffered from benign lesions and 4 (19%) from malignant lesions. Symptoms were mostly nonspecific, with pain being the most common (11/21 (52%)). Diagnosis was incidental in eight cases. Magnetic resonance imaging was performed in 20 (95%) and biopsy was obtained in 10 (48%). Twenty patients underwent surgery, mostly via a posterior approach (14/20 (70%)). At a mean follow-up of 42 months (median 10 months, range 1–166 months), the local recurrence rate was 19%. There was no mortality. Our Pubmed search identified 39 publications.

**Conclusion:**

Our data confirms the significant heterogeneity of retrorectal tumors, which poses a challenge to management, especially considering the often nonspecific symptoms. Regarding diagnosis and treatment, our data highlights the importance of MRI and surgical resection. In particular a malignancy rate of almost 20% warrants a surgical resection in case of the findings of a retrorectal tumour. A local recurrence rate of 19% supports the need for follow up.

## Introduction

Retrorectal tumors are rare and heterogeneous, with an estimated incidence of one in every 40,000 hospital admissions [1–3].

The retrorectal space is confined by the rectum with its mesorectal fascia anteriorly, the parietal pelvic fascia posteriorly and the peritoneum cranially. The distal end of the retrorectal space is defined by the fusion of the presacral, parietal pelvic fascia and mesorectal fascia, which cover the levator ani muscle. The parietal fascia separates the retrorectal space from the presacral space. The iliac vessels and ureters are located laterally [2, 4, 5].

The histologic diversity of retrorectal tumors with benign or malignant lesions results from the embryologic development, during which endo-, meso-, and ectodermal tissues undergo modifications. Tumors can be related to any of these. Retrorectal tumors are commonly categorized as congenital, neurogenic, osseous, inflammatory and miscellaneous [6–8].

Most tumors remain asymptomatic or present with nonspecific symptoms and are often diagnosed incidentally [3]. Occasionally, they present as a palpable mass on digital rectal examination. Clinical examination, computed tomography (CT) and magnetic resonance imaging (MRI) are considered the gold standard for preoperative evaluation [1, 9–11]. Determining the exact anatomical location is essential to the surgical approach. A biopsy can be considered, although its value remains controversial [8, 12]. Retrorectal tumors should be completely resected, even if they are asymptomatic.

Due to their rarity and diverse clinical presentations, diagnosing and treating retrorectal tumors remain challenging. We share our 20-year experience with 21 patients, comparing it with existing literature.

## Patients and methods

In our retrospective study, we identified 21 patients treated for retrorectal tumor at the Surgical Department of the University hospital Erlangen between 2002 and 2022. Of these, 20 underwent surgery. We analyzed demographic characteristics (i.e., age, gender), symptoms, diagnosis, treatment (i.e., surgical approach, resection of bone structure), postoperative complications (Clavien-Dindo classification [13]), histopathology and local recurrence. Data were obtained retrospectively from the patient record.

**Fig. 1:**
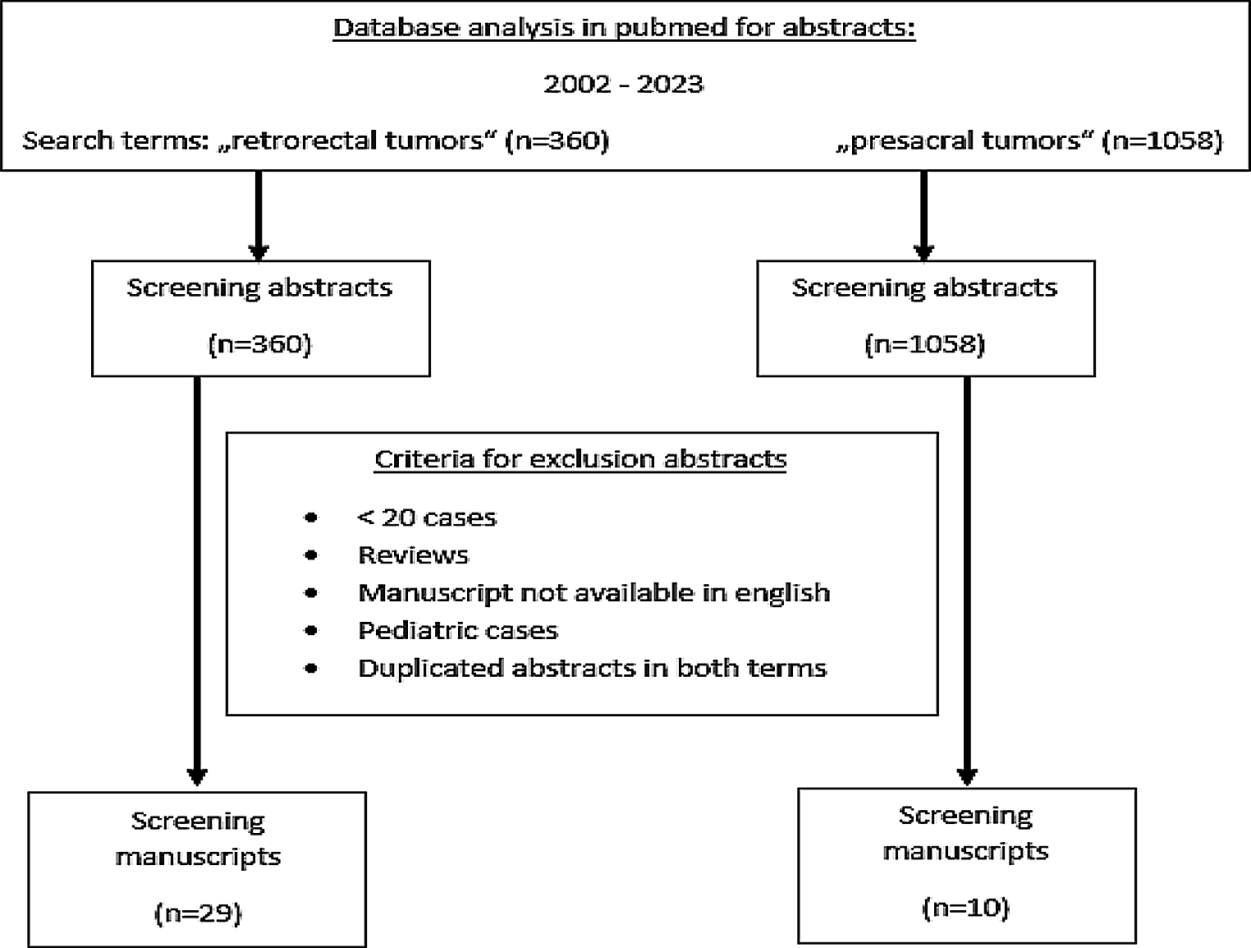
Overview of literature search

Furthermore, in a literature review (Fig. 1), we performed a Pubmed search on January 6, 2023 for abstracts from 2002 to 2023 with the terms “retrorectal tumors” (*n* = 360) and “presacral tumors” (*n* = 1058). Publications with fewer than 20 cases were excluded, as were reviews, manuscripts not available in english, pediatric cases and duplications in both search terms. The remaining publications (*n* = 39) were scrutinized and data extracted to tabulate the findings according to publication year, number of patients, gender, age, histopathology, rate of malignancy, surgical approach, postoperative morbidity, follow-up and local recurrence.

## Results

Patient characteristics are presented in Table 1. Thirteen were female (62%) and eight male (38%). The median age at operation (or, in the non-operated patient at first diagnosis) was 54 years (range 19–74 years). Thirteen patients (62%) presented with nonspecific symptoms: pain in the back, flank, pelvis, lower abdomen, anus or a feeling of anal pressure. One patient had right-sided weakened foot dorsiflexion. Eight patients (38%) were diagnosed incidentally during gynecologic examination, treatment of anal fistula, on MRI or CT for other reasons, and during a gynecologic operation.

**Table 1:** Characteristics and diagnostics of the 21 patients with retrorectal tumor

Patient demographics	
Age (years), median (range)	54 (19–74)
Gender, n (%)	
Female	13 (62)
Male	8 (38)
Diagnostics, n (%)	
MRI	20 (95)
Rectoscopy/Colonoscopy	17 (81)
CT	9 (43)
Biopsy	10 (48)
Endosonography	7 (3)

**Fig. 2:**
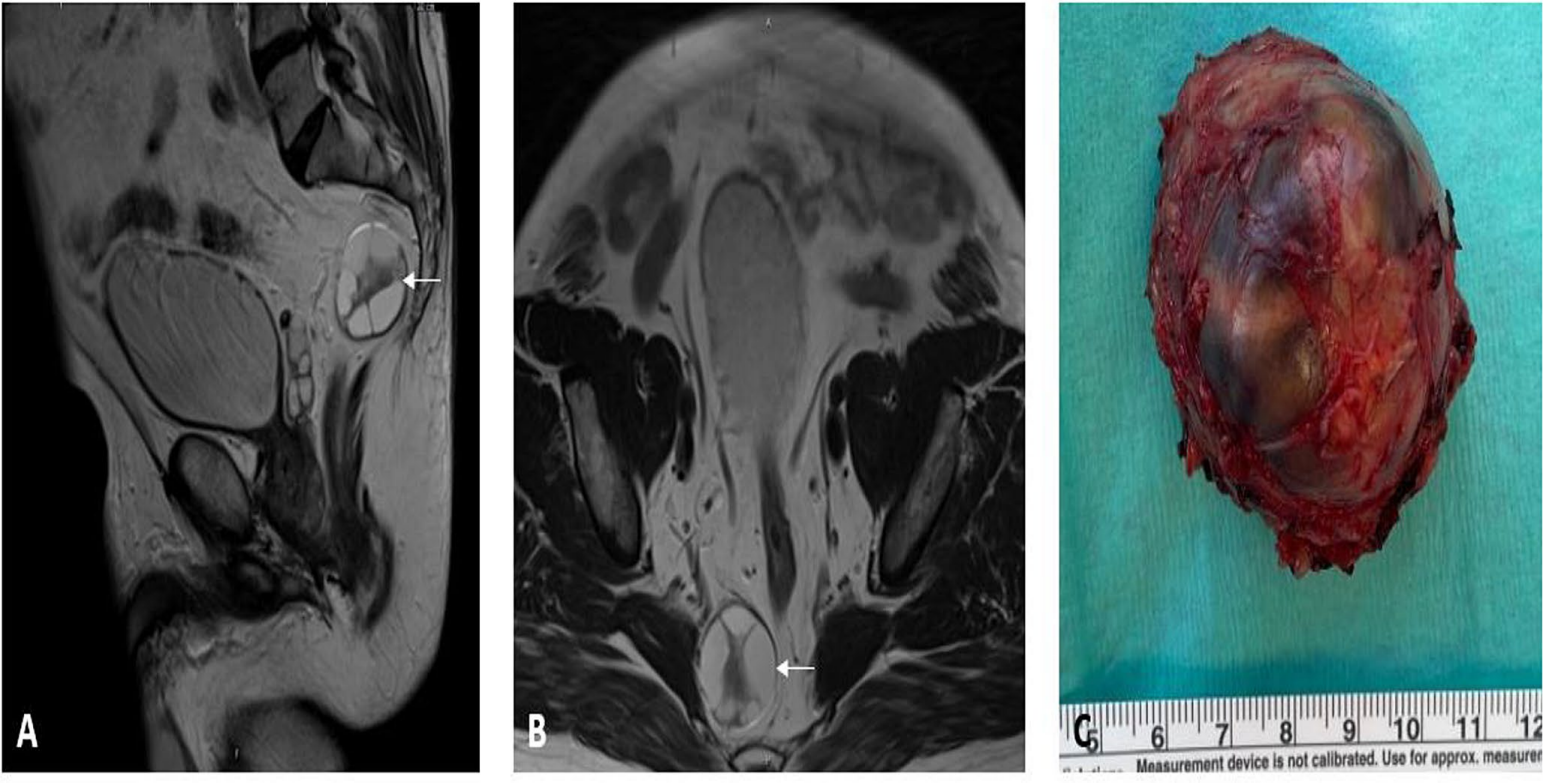
58-year-old patient with a retrorectal schwannoma on preoperative MRI:  sagittal T2 (**A**),  axial T2 (**B**), and the postoperative macroscopic view of the schwannoma (**C**)

MRI was performed in 20 (95%) patients (Fig. 2), CT scan in 9 (43%), endosonography in 7 (33%) and rectoscopy or colonoscopy in 17 (81%). Biopsies were obtained in 10 (48%).

### Treatment

Twenty patients underwent surgery (Table 2); one patient with choroidal melanoma metastasis (diagnosis confirmed by biopsy) underwent radio- and immunotherapy.

**Table 2:** Surgical treatment and outcome of the 21 patients with retrorectal tumor

Surgery and outcome	
Surgical resection performed, n (%)	20 (95)
Surgical approach (*n* = 20)*, n (%)	
Posterior approach (Kraske)	14 (70)
Anterior approach	5 (25)
Combined approach	1 (5)
Additional bone resection (*n* = 20)*, n (%)	9 (45)
Postoperative complication (*n* = 20)*, n (%)	
Clavien-Dindo I	4 (20)
Clavien-Dindo II	1 (5)
Clavien-Dindo III	2 (10)
Postoperative mortality (*n* = 20)*, n (%)	0 (0)
Local recurrence, n (%)	4 (19)

* only patients with surgical resection

A posterior approach (Kraske procedure) was used in 14 patients (70%) and an anterior approach in five (25%). A combined approach was required in one patient (5%). Resection of bone structures was necessary in nine (45%).

Postoperative complications occurred in seven patients (35%): three with wound healing disturbances and one each with a voiding dysfunction, a wound seroma, a hematoma and constipation. All seven patients reported pain. According to the Clavien-Dindo classification, category I occurred in four patients (20%), II in one (5%) and III in two (10%). Four patients had a local recurrence during a median follow-up of 10 months (range 1-166 months) and a mean follow up of 42 months. Reoperation was not required. There was no mortality observed.

### Histopathologic findings

Histopathologic findings varied widely (Table 3). Seventeen patients (81%) had a benign lesion, the most common being a tailgut cyst in 10. In one patient it remained unclear whether the lesion was a tailgut or a duplication cyst. Schwannoma was diagnosed in three cases and an osseous pseudotumor, a lipoma and a teratoma in the other three.

**Table 3:** Histopathological findings

Histopathological findings	
Benign tumor, n (%)	17 (81)
Tailgut cyst	10 (48)
Tailgut cyst DD Duplication cyst	1 (5)
Schwannoma	3 (14)
Osseous pseudotumor	1 (5)
Lipoma	1 (5)
Teratoma	1 (5)
Malignant tumor, n (%)	4 (19)
Solid fibrotic tumor (Hemangiopericytoma)	1 (5)
Metastasis	1 (5)
Eosinophil chordoma	1 (5)
Adenocarcinoma in a tailgut cyst	1 (5)

*DD = differential diagnosis*

Four tumors were revealed to be malignant (19%): a mucinous adenocarcinoma in a tailgut cyst, a choroidal melanoma metastasis, a solid fibrotic tumor (hemangiopericytoma) and an eosinophil chordoma.

### Literature search

In the 39 publications, the recorded characterics regarding number of patients, gender, age, histopathology, rate of malignancy, surgical approach, postoperative morbidity, follow-up, and local recurrence are presented in Table 4.

**Table 4:** Overview of literature

No.	Author	Year	No. of patients	Woman: Man	mean age (range)	Histopathology	Rate of malignancy	Surgical approach	Postoperative morbidity	Follow-up median months – (range)	Local recurrence
1	Mualem et al.[14]	2022	27	12: 15	49,9 ± 11,5 (33–71)	spinal schwannoma		Anterior 6: Posterior 15: Combined 4	4 (14,8%)	37,1* ± 62,1 (0–228)	7
2	Zhao et al.[15]	2022	110	82: 28	40,0 ± 13,2 (12–69)	variable	13 (11,8%)	Posterior (transsacrococcygeal transverse incision) 105: Anterior (transabdominal excision) 1: Combined 4	4 (3,6%)	90,13* ± 31,22 (35–155)	11 (10%)
3	Broccard et al.[9]	2022	73	58: 15	44,4* (18–81,2)	tailgut cysts	6 (8,2%)	Parasacral 56 (76,7%): Transabdominal 13 (17,8%): Combined 4 (5,5%)	18% (at 30 days)	4,5	3 (4,1%)
4	Burke et al.[16]	2022	144	103: 41	49* (37–61 i.q.r.)	variable	25 (17,3%)	Transperineal 76 (52,7%): Abdominal 59 (40,9%): Combined 9 (6,2%)	33 (22,9%)	82 (47–126 i.q.r.)	19 (13,2%)
5	Aubert et al.[17]	2021	270	213: 57	46 ± 15 (18–77)	variable	8%	Abdominal 72 (27%): Bottom 190 (70%): Combined 8 (3%)	81 (30%)	27* ± 39 (1–221)	8%
6	Gould et al.[18]	2021	143	106: 37	46*	variable	17 (11,9%)	Posterior/perineal 64 (60%): Open trans-abdominal 14 (13%): Laparoscopic 10 (9%): Combined 15 (14%): Transanal 1 (1%): Transvaginal 1 (1%): Transvaginal 1(1%): Drainage of lesion only 2 (2%)	n.a.	n.a.	14 (27%)
7	Zhang et al.[19]	2021	122	95: 27	34* (18–81)	variable	21 (17,2%)	Transabdominal 9: Posterior 99: Combined 14	19 (postoperative complications)	malignant tumors: 45 (14–101), benign tumors: 49 (12–119)	1 (5%)
8	Li et al.[20]	2021	44	33: 11	50* (13–87)	variable	18 (40,9%)	Posterior 26 (83,9%): Anterior 3 (9,7%): Combined 2 (6,5%)	n.a.	25 (3–93)	9 (37,5%)
9	Carpelan-Holmström et al.[21]	2020	52	40: 12	43* (19–76)	variable	4 (8%)	Perineal 44 (85%): Abdominal 7 (13%): Combined 1 (1,9%)	11 (21%)	n.a.	14 (27%)
10	Yalav et al.[22]	2020	20	12: 8	48,3 ± 14,2	variable	3 (15%)	Posterior 14 (70%): Anterior 2 (10%): Combined 4 (20%)	7 (35%)	53,8* ± 40	1 (5%)
11	Houdek et al.[23]	2020	65	42: 23	58 (27–81)	chordoma	100%	“sacrectomy” (all patients)	44 (67,7%)	n. a.	n. a.
12	Zhou et al.[24]	2020	20	19: 1	36* (22–64)	variable	in teratomas: one contained focal mucinous adenocarcinomas, another three had components of low-grade mucinous neoplasm	Laparoscopic 17: Combined laparoscopic - posterior 3	3	36 (6–64)	1 (5%)
13	Sakr et al.[25]	2019	24	18: 6	51,5* (21–68)	tailgut cysts	2 (8,3%)	Anterior, laparoscopic 10 (41,7%): Posterior 11 (45,8%): Combined 3 (12,5%)	10 (41,7%)	12 (1–66)	0
14	Poškus et al.[26]	2019	35	29: 6	49,88 ± 14,48	variable	n.a.	Laparotomy 19: Perineal 11: Combined 1: Laparoscopic 3	6 (17,14%)	71,83*	0
15	Dziki et al.[27]	2019	29	13: 16	48 (19–80)	variable	10 (34%)	Transsacral 15 (51%): Abdominal laparotomy 12 (41%): Combined 2 (7%)	6 (21%, up to 30 days); long-term complications 10 (34%)	48*	11% (benign lesion); 40% (malignant lesion)
16	Dwarkasing et al.[28]	2017	28	22: 6	range 18–70	variable	5 (18%)	n.a.	n.a.	n.a.	n.a.
17	Maddah et al.[29]	2016	50	26: 24	41,7 (16–74)	variable	21 (56,7%)	Laparotomy 11: Sacral 17: Anterior-posterior 14: Abdominal-sacral 1	8	56,7* (10–277)	n. a.
18	Buchs et al.[30]	2016	62	50: 12	44,2 ± 14,7 (20–76)	variable	13 (21%)	Posterior 55 (88,7%): Abdominal or combined 7 (11,3%)	n. a.	36,9* ± 33,7 (1–132)	9 (14,5%)
19	Sun et al.[31]	2016	64	29: 35	37,2 (21–69)	sacral neurogenic tumors: 38 neurilemmomas, 26 neurofibromas		Anterior 19: Posterior 25: Combined 20	n. a.	58,2* (24–93)	8 (12,5%)
20	Hopper et al.[32]	2016	69	42: 27	50* (36–67 i.q.r.)	variable	20 (29%)	Posterior 15 (55%): Combined abdominoperineal 6 (22%): Anterior 3 (11%)	n. a.	20 (5–66 i.q.r.)	n. a.
21	Gong et al.[33]	2015	36	29: 7	42 ± 14,4 (18–69)	variable	n. a.	Transcoccygeal (all)	n. a.	n. a.	n. a.
22	Simpson et al.[34]	2014	26	19: 7	37,5* (16–76)	sacrococcygeal teratoma	5 (19,2%)	Posterior 15: Anterior 5: Combined 6	61% (30-day complications)	benign tumors: 23 (0,33–396)	1 (3,8%)
23	Sagar et al.[35]	2014	76	50: 26	48* (19–88)	variable	16 (21%)	Abdominal 41: Transperineal 31: Combined abdominoperineal 4	n. a.	n. a.	n.a.
24	Messick et al.[36]	2013	87	67: 20	44* (19–88)	variable	23 (26%)	Posterior 50 (60%): Anterior 25 (30%): Combined anteriorposterior 9 (10%)	n. a.	8 (0,1–225)	11% (7/64) of benign tumors
25	Chéreau et al.[37]	2013	47	34: 13	45,8 (17–85)	variable	9 (19%)	Perineal 42 (89%): Abdominal or combined 5 (11%)	4 (9%)	71 (2–168)	n. a.
26	Macafee et al.[38]	2012	56	37: 19	51* (20–88)	variable	17 (37,5%)	Abdominal 27 (48%): Perineal/transsacral 20 (36%): Composite abdomino-sacral 9 (16%)	19	46 (6–90)	2
27	Du et al.[39]	2012	93	61: 32	41,4* (15–71)	variable	21 (22,6%)	Transsacral 78: Transabdominal 12: Combined 3	n. a.	n. a.	n. a.
28	Li et al.[40]	2011	33	13: 20	48,5 (18–71)	variable	4 (12,1%)	Transabdominal 10 (30%): Transsacral 18 (55%): Combined abdomino-sacral 5 (15%)	5 (15%) wound complications + 4 (12%) sphincter disfunction	45,1* (14–123)	4 (12%)
29	Lin et al.[41]	2011	62	39: 23	40,5* (15–68)	variable	14 (22,6%)	Transsacral 52: Transabdominal 8: Combined 2	n. a.	n. a.	7 (of 45 cases)
30	Gao et al.[42]	2011	39	29: 10	39,56 (14–71)	variable	6 (15,38%)	Transsacral 26: Transabdominal 8: Combined 2	8 (perioperative complications)	47 (4–110)	2 (5%)
31	Dozois et al.[43]	2011	37	17: 20	49* (22–81)	sarcomas	100%	n. a.	21 (58%)	56,4 (in 16 patients alive)	n. a.
32	Yang et al.[44]	2010	21	13: 8	39,3 (16–74)	variable	7 (33,3%)	n. a.	n. a.	n. a.	n. a.
33	Mathis et al.[45]	2010	31	28: 3	52* (27–79)	tailgut cysts	4 (12,9%)	Posterior 20: Anterior 9: Combined 2	8 (25,8%)	2,0 years (1,0–22,6 years): *n* = 16	1 (3,2%)
34	Wei et al.[46]	2009	48	30: 18	47 (17–75)	sacral neurogenic tumors	7 (14,6%)	Anterior 7: Posterior 22: Combined 19	n. a.	47* (20–96)	5
35	Pappalardo et al.[47]	2009	34	19: 15	42 (14–75)	variable	14 (41%)	Transsacral 1: Transperineal 6: Perineal 1: Parasacral 4: Mixed posterior & anterior contemporary 6: Mixed posterior & anterior sequential 4: Laparotomy 7: Posterior 3: Abdomino-perineal 2	n. a.	n. a.	n. a.
36	Grandjean et al.[48]	2008	30	23: 6	43 (16–77)	variable	1 (3,3%)	Transanal 3: Posterior 23: Anterior 2: Combined posterior & anterior 2	6 (20%)	38,4* (6–180)	2 (7%)
37	Woodfield et al.[49]	2008	27	17: 10	Benign 30* (21–88), Malignant 60* (47–77)	variable	7 (25,9%)	Perineal 12: Abdominal 11: Combined 4	3 (11%)	benign tumors: 49 (2–72), malignant tumors: 26 (10–61)	3 (11%)
38	Glasgow et al.[50]	2005	34	21: 13	48 (21–80)	variable	7 (21%)	Anterior 14: Posterior 11: Combined abdominoperineal 9	n. a.	benign tumors: 22	n. a.
39	Lev-Chelouche et al.[51]	2003	42	28: 14	40,6 (21–84)	variable	21 (50%)	Anterior 18: Posterior 21: Combined 3	15 (36%)	benign congenital tumors: 54 (2–94), malignant congenital tumors: 22 (4–118), benign acquired tumors: 27, malignant acquired tumors: 49* (15–103)	12 (28,6%)

Age* = median age; ± standard deviation; i.q.r.= interquartile range; follow-up*= mean follow-up

## Discussion

This study represents a single-institution series of retrorectal tumors and demonstrates a heterogeneity comparable to other reports and few systematic reviews [1–4, 9, 52, 53]. Its reported incidence ranges from 0.9 to 6.3 patients per year and is estimated as one in 40,000 hospital admissions [1, 3, 4, 52]. In our retrospective study, we report on 21 patients treated between 2002 and 2022.

Retrorectal tumors can be divided into five categories, congenital (55–65%) being the most common [1, 4, 6–8, 54]. As in our data the vast majority are benign and occur predominantly in females. Two of the four malignant tumors in our series, however, were found in male patients.

During embryologic development, a tail is formed from the endo-, meso-, and ectodermal tissues. If the tailgut does not recede, a remnant can result as a tailgut cyst [3, 6]. Resection is recommended because of the risk of malignant transformation [9, 55]. In our study, in accordance with published data [9, 55], benign tailgut cysts were the most common entity, while in one patient a poorly differentiated mucinous adenocarcinoma was found in the cyst. In another patient it remained unclear whether the lesion should be classified as a tailgut or duplication cyst.

The rate of malignancy is reported to range up to 26.6% [9, 56]. In 2022, Burke et al. described a malignancy rate of 17.3% in a large series of 144 tumors [16], which accords with our data. The highest rate of neoplasia of 26.6% was found in a systematic review comprising 196 patients [56].

The most frequent malignant retrorectal tumor is the chordoma, which results from persistence of endoderm, probably from residue of the chorda dorsalis [1, 8, 57]. In our study one patient presented with an eosinophilic chordoma.

With a frequency of 10-12%, neurogenic tumors are the second most common entity and are predominantly benign [4]. In various publications, schwannomas, in particular, have been described, as we found in our study (see Fig. 2) [1, 58].

Another 12–16% of retrorectal tumors are miscellaneous, often rare entities [3, 4, 7, 8]. In single patients, we found benign lesions (osseous pseudotumor, lipoma) as well as a malignant lesion with a solid fibrotic tumor (hemangiopericytoma), and a previously unreported metastasis of a choroidal melanoma.

The presentation can be nonspecific, even asymptomatic, and thus diagnosis is often incidental and at an advanced stage [1]. Indeed, the majority of our patients had nonspecific symptoms such as back and lower abdominal pain, and diagnosis was based on incidental findings in one quarter. Neurologic symptoms, such as the dorsiflexion of the foot seen in one, can also occur.

MRI and CT scans are considered the gold standard for evaluating these tumors beside the clinical examination. MRI can distinguish tissue properties and relations to neighboring organs [1, 6], often allowing accurate tumor diagnosis. In our series, 95.2% of the retrorectal tumors were confirmed or detected by MRI. CT allows clear visualization of bone structures and the differentiation between solid and cystic lesions [1, 6].

The use and value of biopsy remains controversial in current literature [1, 6, 12, 53]. Glasgow and Dietz refer to the risk of infection with subsequent sepsis, such as a biopsy of an anterior sacral meningocele leading to meningitis [8]. Additionally, the risk of biopsy-related tumor cell dissemination has to be considered. If tumor categorization is not possible and the option of neoadjuvant therapy must be considered, biopsy appears to be reasonable. In our study, in 47.6% of cases a biopsy was performed. It proved to be essential to therapeutic planning (radiation and immunotherapy) in the patient with the choroidal melanoma metastasis, our only patient not undergoing surgery.

Treatment depends on the tumor entity. In most cases - including asymptomatic tumors - a complete resection is indicated because of the potential for tumor growth with increasing symptoms and risk of malignant transformation [8, 52].

For surgical planning the location and size of the tumor and its relationship to neighbouring organs are relevant. Diagnostic and therapeutic algorithms have been proposed [11, 18, 32, 49]. Surgical options are posterior, anterior or combined approaches. As described by Dozois et al. [59, 60], a line through sacral vertebra three is helpful for decision-making. For small tumors below this line, the posterior approach may be sufficient, like that first described by Kraske in 1886 as the transcoccygeal approach for rectal cancer [8, 61]. This is the most common approach and was used In 70% of our patients. If the tumor is above the S3 line, the anterior, abdominal approach is advisable, although large tumors may require a combined approach. In 45% of our patients, a resection of bone structures (e.g. the os coccygis) became necessary to facilitate operative access or achieve complete tumor resection.

The postoperative complications in seven patients were Clavien-Dindo classification I in most (*n* = 4 (20%)) and were comparable to other studies [3, 13, 52]. In four patients a local recurrence was diagnosed. With no mortality, the resection of retrorectal tumors proved a predominantly safe procedure.

This study represents a comprehensive single-institution series of retrorectal tumors. The relatively small number of patients in this study likely may owe to the the rarity of retrorectal tumors. The retrospective design may affect accurate representation of the recurrence rate, however the represented rate of recurrence support the idea of a follow-up.

## Conclusion

Retrorectal tumors are a heterogeneous entity. Our data show that most are benign. Resection is recommened and malignant entities may require multimodal therapy. In our cohort one patient had a very rare retrorectal metastasis of a choroidal melanoma, and another had a mucinous adenocarcinoma in a tailgut cyst. Biopsy may be helpful with inconclusive MRI findings and solid tumors. Decision-making by an interdisciplinary tumor board is recommended. The choice of surgical approach is determined by the tumor’s location and size. In our series, the posterior approach was most frequent.

## Data Availability

The datasets generated during the current study are available from the corresponding author on reasonable request, but are not public due to privacy restrictions, as they were obtained from medical records.
